# Immunoinformatics approaches in developing a novel multi-epitope chimeric vaccine protective against *Saprolegnia parasitica*

**DOI:** 10.1038/s41598-024-52223-z

**Published:** 2024-01-27

**Authors:** Abhigyan Choudhury, Pawan Kumar, Hiba-Allah Nafidi, Khalid S. Almaary, Gezahign Fentahun Wondmie, Ajit Kumar, Mohammed Bourhia

**Affiliations:** 1 Independent Researcher, Asansol, West Bengal 713 305 India; 2 Toxicology and Computational Biology Group, Centre for Bioinformatics, Maharshi Dayanand University, Rohtak, 124 001 India; 3 Department of Food Science, Faculty of Agricultural and Food Sciences, Laval University, Quebec City, QC 2325G1V 0A6 Canada; 4 Department of Botany and Microbiology, College of Science, King Saud University, P. O. Box 2455, 114 51 Riyadh, Saudi Arabia; 5 Department of Biology, Bahir Dar University, Po.Box 79, Bahir Dar, Ethiopia; 6 Department of Chemistry and Biochemistry, Faculty of Medicine and Pharmacy, Ibn Zohr University, 700 00 Laayoune, Morocco; 7 Laboratory of Chemistry-Biochemistry, Environment, Nutrition, and Health, Faculty of Medicine and Pharmacy, University Hassan II, B. P. 5696 Casablanca, Morocco

**Keywords:** Biotechnology, Computational biology and bioinformatics, Drug discovery

## Abstract

*Saprolegnia parasitica* is responsible for devastating infections in fish and poses a tremendous threat to the global aquaculture industry. Presently, no safe and effective control measures are available, on the contrary, use of banned toxic compounds against the pathogen is affecting humans via biomagnification routes. This pioneering study aims to design an effective multi-epitope multi-target vaccine candidate against *S. parasitica* by targeting key proteins involved in the infection process. The proteins were analyzed and linear B-cell epitopes, MHC class I, and class II epitopes were predicted. Subsequently, highly antigenic epitopes were selected and fused to a highly immunogenic adjuvant, 50S ribosomal protein L7/L12, to design a multi-epitope chimeric vaccine construct. The structure of the vaccine was generated and validated for its stereochemical quality, physicochemical properties, antigenicity, allergenicity, and virulence traits. Molecular docking analyses demonstrated strong binding interactions between the vaccine and piscine immune receptors (TLR5, MHC I, MHC II). Molecular dynamics simulations and binding energy calculations of the complexes, further, reflected the stability and favorable interactions of the vaccine and predicted its cytosolic stability. Immune simulations predicted robust and consistent kinetics of the immune response elicited by the vaccine. The study posits the vaccine as a promising solution to combat saprolegniasis in the aquaculture industry.

## Introduction

Oomycetes, also called water moulds, are filamentous organisms belonging to the kingdom Chromista that pertain fungus-like lifestyles. They are found widely in freshwater and marine habitats as well as on plant surfaces and are well known for their capacity to cause a range of diseases in plants and animals, including fish, crustaceans, and amphibians. One such notorious species among this class is *Saprolegnia parasitica.* It infects fish eggs resulting in instant mortality while also infecting adult fishes via epidermal invasion and conducting a progressive and lethal infection. The infection is most commonly indicated by the presence of fluffy, white or greyish cotton-like tufts on the skin, fins, and gills. These tufts are composed of filaments that penetrate the fish tissues, causing tissue damage, inflammation, and necrosis. Infected fish may exhibit behaviours such as lethargy, loss of appetite, and increased susceptibility to other infections before it succumbs to death upon progression of the disease^1–4^. The target fish population of *S. parasitica* is found in both tank and wild environments and it is endemic to all the water habitats in the world. It has a tremendous impact on global fish health, population, and fish-based economy. A wide number of investigations suggest that such infections have been a leading cause behind the reduction in survivability of migratory like salmons and trouts^5–7^. Its ability to invade tank fish populations has implications ranging from deliberately disrupting laboratory research setups to economically destabilizing the worldwide aquaculture industry^8^. In order to deter such calamities, Malachite Green, a dye compound found its use as an anti-*Saprolegnia* agent. However, a worldwide ban was enforced on it soon after its toxicological and carcinogenic effects were revealed. Nevertheless, Malachite Green, being financially inexpensive, is now seeing illegal use in several aquaculture sectors that is ultimately resulting in biomagnified toxicity to man^9–14^. As of now no safe and effective control regimen could have been proposed against this pathogen and this study aims to change the same.

**Figure 1:**
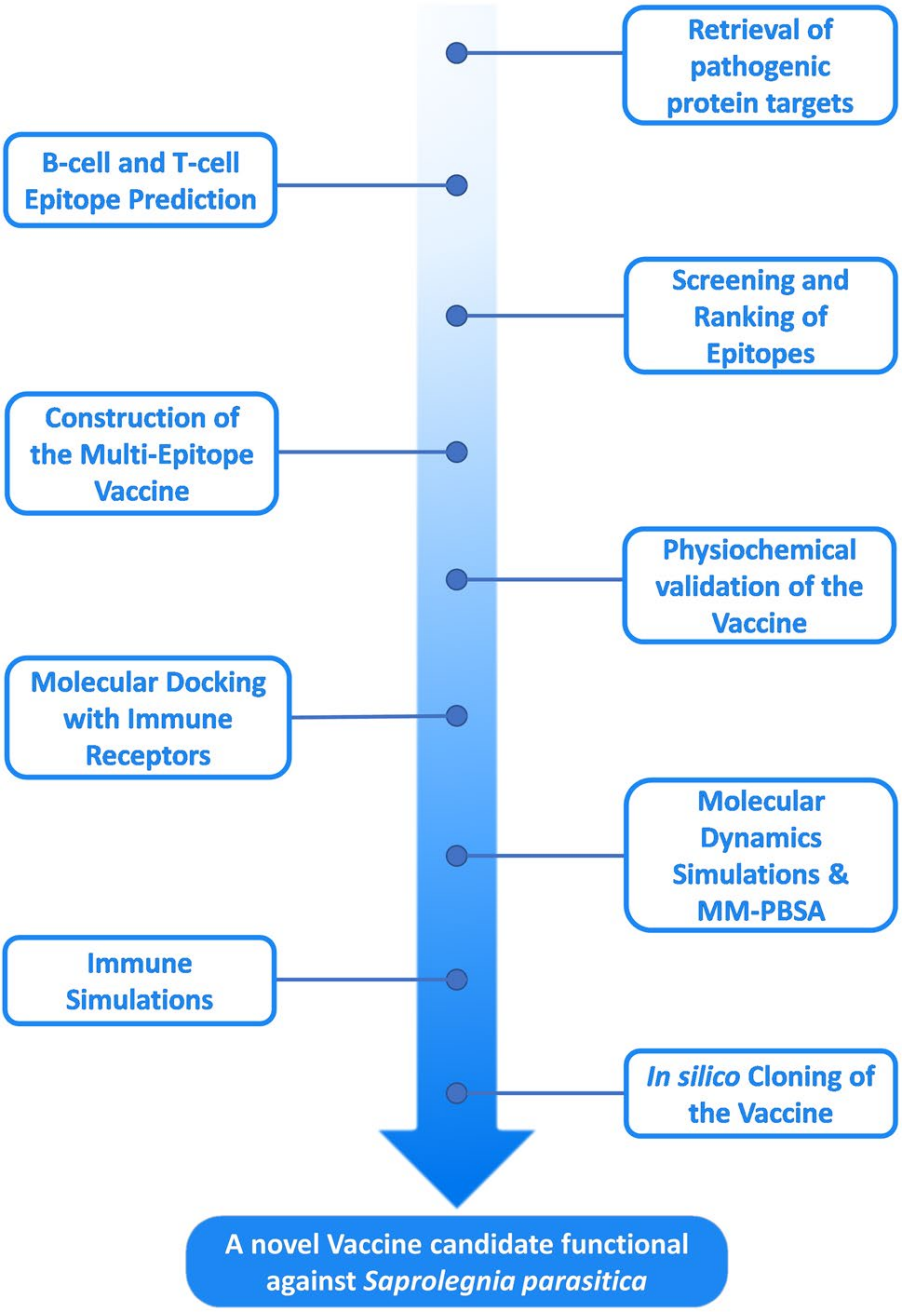
Sequential flow of reverse vaccinology strategy in design a novel vaccine candidate against *S. parasitica*.

*Saprolegnia parasitica* has an elaborate life cycle among which the asexual stages determine its pathogenicity^15,16^. Sporulation and dispersal of the primary zoospores from the hyphae initiates the first phase of Saprolegniasis i.e. infection of the primary host fish. Alternatively, the primary zoospores might encyst inside the host and form primary cysts and later on release secondary zoospores that are considered as the main infective stage. This initiates a series of infections to different fish hosts and consequent formation and dispersal of secondary zoospores, thus, gaining infectivity to a broader host population with each generation. The preliminary *S. parasitica*-host fish interactions involve the secretion of virulence factors called effector proteins by it into the extracellular matrix of the fish cells and initiate the process of infection^17,18^. One such key protein is *S. parasitica* host targeting protein 1 (SpHtp1) which is secreted in the pre-infection and early stages of the infection, and is a RxLR motif (whereby “x” can be any amino acid) containing protein that translocates into the host epidermal cell cytoplasm via tyrosine-O-sulfate dependent manner. Once inside the cell, it triggers a signalling cascade that leads to the internalization of the pathogen. Likewise, another protein named *S. parasitica* host targeting protein 3 (SpHtp3) is secreted, however, unlike SpHtp1 it doesn’t use RxLR motif for translocation instead its C-terminal interacts with GP96-like proteins present in the lipid rafts of the membranes of the fish epidermal cells. SPRG 19320 is a putative Thrombospondin type 1 domain containing protein that composes a major part of the secretome of *S. parasitica* and has been evident in all the life stages of the organism^19–22^. Hence, any therapeutic intervention at this secretome level during the early infection stages can offer a significant impact in terms of prophylaxis. In essence, biocomputational methods have played a crucial role in the investigation of potential inhibitory mechanisms targeting *S. parasitica*^23,24^. Nonetheless, it is important to note that only a vaccine can effectively establish herd immunity against this pathogen. The development timelines for vaccines were approximately 25 years for varicella, 5 years for Ebola, and just 1 year for COVID-19, evidently, immunoinformatics approaches have been revolutionary in the field of vaccine design. Not only it reduces the time and cost of the development process drastically but also elevates the efficacy and safety of the vaccine by using the reverse vaccinology techniques^25^. The present study employs similar techniques and seeks immunogenic characterization of the aforementioned key proteins in order to design a novel vaccine that promises to eradicate the present threat of Saprolegniasis to the aquaculture industry (Fig. 1).

## Materials and methods

### Sequence retrieval

Being the first step in the development of the vaccine, the SpHtp1, SpHtp3 and SPRG 19320 proteins were strategically selected considering their instrumental roles in the infection pathology of *S. parasitica* and the predominant presence in the secretome, subsequently, their amino acid sequences were extracted from GenBank database sequence files having accession ID. ADB84848.1, A0A067CMC7, and KDO33710.1 respectively.

### B-cell epitope screening

B-lymphocytes have been prime conductors of humoral immunity and are responsible for producing antibody molecules for detecting and neutralizing the pathogenic antigens. The retrieved protein sequences were scanned for linear B-cell epitopes using Kolaskar and Tongaonkar algorithm from the Antibody epitope prediction tool at the Immune Epitope Database (IEDB) analysis server (http://tools.iedb.org)^26^. The server acts as a centralized database that houses a wide range of epitope-related information relevant to multiple immune processes, such as vaccine development, infectious diseases, autoimmune conditions, and cancer immunotherapy.

### Epitope mapping for MHC class I and II presenter compatibility

Cytotoxic T-cells are crucial for producing MHC-I based responses as they destroy the host cells infected by *S. parasitica*. Moreover, MHC II enabled helper T-cells play a very important role in adaptive immunity by stimulating B-cells and cytotoxic T-lymphocytes for subsequent production of antibodies and destruction of infected or damaged host tissue. NetMHC v4.0 (https://services.healthtech.dtu.dk/services/NetMHC-4.0/)^27^ server was used to predict the MHC class I epitopes while NetMHCII v2.3 tool (https://services.healthtech.dtu.dk/services/NetMHCII-2.3/)^28^ screened the proteins for MHC II epitopes.

### Antigenicity assessment

Epitopes predicted were enormous in number but not all epitopes possess equivalent capacity to generate immunogenic responses, hence, the antigenicity of the predicted pathogenic epitopes was evaluated using the VaxiJen 2.0 server (http://www.ddg-pharmfac.net/vaxijen/)^29,30^ that uses Auto Cross-Covariance (ACC) transformation algorithm to find out the antigenic strength of the epitopes right from their sequences. Consequently, highly antigenic epitopes against *Saprolegnia* targets were chosen.

### Structural construction and validation of the vaccine candidate

In order to develop an effective vaccine, it is crucial to consider the proper folding and presentation of antigenic peptides, ensuring accessibility to immune cells while maintaining the stability of the vaccine’s protein structure. To achieve this, the design incorporates linkers to join the epitopes within the structure. Linkers have a crucial role in minimizing junctional immunogenicity and preserving the identity of individual epitopes during vaccine processing within cells, thus, ensuring the immunogenicity of each epitope. A multi-epitope vaccine lacking linkers may lead to the creation of a new protein with unknown properties or the formation of neoepitopes or junctional epitopes^31^. The construct peptide starts with a highly immunogenic adjuvant, the 50S ribosomal protein (L7/L12) (Accession ID: P9WHE3), which is linked to a series of MHC I epitopes using an EAAAK linker. This linker is a rigid α-helix forming peptide linker that offers efficient separation of the functional domains of the adjuvant and the epitopes by keeping a fixed distance with minimal interference from the epitopes thereby maintaining their individual functional properties^32,33^. On the other hand, KK linkers were employed to connect the B-cell epitopes as these Lysine linkers are the target for the Cathepsin B, a lysosomal protease involved in processing of the antigenic peptides for their presentation on the cell surface. It also plays a crucial role in reducing the junctional immunogenicity by avoiding the induction of antibodies for the peptide sequence that individual epitopes can form when they are joined linearly^34,35^. Likewise, the AAY (Ala-Ala-Tyr) linker is the cleavage site for the proteasomes in mammalian cells. Hence, the linkers were used to join the MHC-I epitopes such that they cleave and separate effectively within the cells^36,37^. The MHC-II epitopes were conjugated using GPGPG linkers as they not only disrupt the junctional immunogenicity and restore the individual immunogenicity of the epitopes but they are also found to activate helper T-lymphocyte responses^38^. The tertiary structure of the multi-target, multi-epitope vaccine peptide is generated using Robetta employing the RoseTTAFold (https://robetta.bakerlab.org)^39^ algorithm for de novo protein modelling. The generated model undergoes stereochemical validation using the ERRAT program^40^ provided by the SAVES 6.0 server (https://saves.mbi.ucla.edu), the SWISS-Model Structural Assessment tool (https://swissmodel.expasy.org/assess)^41^, as well as the ProSA web server (https://www.came.sbg.ac.at)^42^.

### Physiochemical assessment of the candidate

A comprehensive analysis of the vaccine’s physicochemical properties is imperative after its design. The ExPASy ProtParam tool (https://web.expasy.org/protparam/)^43^ was employed as the primary method to assess the physicochemical characteristics of the vaccine peptide. Additionally, the overall antigenicity of the vaccine was determined using the VaxiJen 2.0 server. To evaluate the allergenic nature of the peptide, the AllerTOP 2.0 tool (https://www.ddg-pharmfac.net/AllerTOP/)^44^ was utilised, while the VirulentPred tool (https://bioinfo.icgeb.res.in/virulent/)^45^ was employed to assess the virulence traits of the vaccine. Also, the Protein–Sol server (https://protein-sol.manchester.ac.uk) was used to assess the solubility of the vaccine in order to evaluate its functionality in the humoral hydrophilic environment^46^. Furthermore, the ElliPro tool (http://tools.iedb.org/ellipro/)^47^ from IEDB was utilized to map the conformational B-cell epitopes of the vaccine, ensuring a thorough understanding of its immunogenic potential.

### Molecular docking with immune receptors

Ensuring stable interactions between the vaccine candidate and immune receptors is a critical prerequisite for the generation of effective immune responses. Toll-like receptors (TLRs) play a pivotal role in both innate and adaptive immunity, serving as a crucial link between the two. Notably, TLR5 has been identified as a key player in recognising fungal pathogens^48–50^. Additionally, class I and class II MHC molecules facilitate the recognition of foreign antigens, including those present in vaccines, by CD4+ and CD8+ T cells. To investigate these interactions, the crystal structure of fish TLR5 (PDB ID: 3V44) was retrieved. While the structure of MHC I’s common fish allele HLA-A*02:01:01:01 was constructed using homology modelling using the SWISS-Model tool, similarly, the structure of common fish MHC class II DAB1*07:01 allele was devised. Subsequently, both these structures underwent validation using the SWISS-Model Structure Assessment tool. Furthermore, the ClusPro 2.0 (https://cluspro.bu.edu/)^51^ supercomputer server was utilised to perform molecular docking operations of the vaccine construct with the fish TLR5, MHC class I, and MHC class II structures, respectively, enabling a comprehensive analysis of their binding interactions. Additionally, the biomolecular structures were visualized using the PyMol software suite ver. 2.5^52^.

### Molecular dynamics simulations

An understanding of how the vaccine polypeptide system would evolve in cytosolic environments while interacting with the immune receptors is essentially required to proceed further with in silico observations. Molecular dynamics (MD) simulations allow us to gain key insights associated with structural stability and, hence, the functionality of the vaccine, in an emulated cytosolic system. In our present study, the piscine vaccine was docked against TLR5, MHC-I, and MHC-II receptors and all the three docked complexes were first pre-processed under the Amber 99SB-ILDN force-field using GROningen MAchine for Chemical Simulations (GROMACS) ver. 2022.4^53,54^. Subsequently, the systems were solvated with the SPCE water model that specifies 3-center water molecules with appropriate charges. Further, Na^+^ and Cl^−^ ions were supplemented to the system at a concentration of 0.9%, tending to imitate natural piscine hemodynamic conditions^55–58^. The entire complex systems were subjected to energy minimization after the pre-processing stage employing the steepest descent algorithm for 5000 steps, each counting 2 fs. Thereafter, in the NVT ensemble, the system was gradually heated from 0 to 298 K within regulation from the Berendsen thermostat. Similarly, in the NPT restraint ensemble, the pressure of the system was adjusted to 1 atm pressure by the Parrinello–Rahman barostat and the equilibration lasted for 50,000 time steps. Finally, all the prepared complexes were subjected to the main 100 ns MD run, wherein snapshots were recorded every 100 ps. The post-simulation analyses in our study involved parameters like Root mean square fluctuation (RMSF), Root mean square deviation (RMSD), Radii of gyration (Rg), Solvent accessible surface area (SASA), along with the Leonard–Jones as well as Coulombic interaction energies in the complexes.

### Binding energy calculations

The binding strength (binding free energy) of the vaccine polypeptide and the studied immune receptors were assessed using Gmx_MMPBSA tool^59^. It describes the energetics of the complexes’ internal interactions; with a continuum solvent model described by the Poisson–Boltzmann equation and a surface area term and can be expressed as following:

$$ \Delta G_{bind,solv} = \, \Delta G_{bind,vaccum} + \, \Delta G_{solv,complex} - \, \left( {\Delta G_{solv, \, ligand} + \, \Delta G_{solv,receptor} } \right). $$

### Immune simulations

The application of immune simulations in modelling the interactions between vaccine antigens and immune cells offers a valuable and efficient means of predicting the magnitude and kinetics of the immune response elicited by the designed vaccine. The C-ImmSim server (www.cbs.dtu.dk/services/C-ImmSim-10.1)^60^ utilizes position-specific scoring matrices (PSSM) derived from machine learning techniques and provides a robust agent-based model for accurately predicting immune interactions. Leveraging this server, a comprehensive simulation spanning 120 days was conducted, wherein a dose of the vaccine peptide was administered during the initial hour, followed by two subsequent doses on the 30th and 60th day, respectively. This systematic approach allows for a detailed assessment of the immune response dynamics and provides insights into the temporal aspects of vaccine efficacy and immunological outcomes.

### Reverse translation and in silico cloning

The manufacturing process of the vaccine candidate is a crucial factor in achieving herd immunity, and it involves the efficient and rapid production of the vaccine through the cloning of vaccine codons into highly optimized expression vectors. To accomplish this, the vaccine peptide sequence was initially reverse translated using the *E. coli* K12 codon table with the assistance of EMBOSS Backtranseq (https://ebi.ac.uk/Tools/st/emboss_backtranseq/), in complementation with the JCat tool (https://www.jcat.de), subsequently, the Codon Adaptation Index (CAI) along with the GC content of the reverse translated sequence were determined^61^. Thereafter, SnapGene ver.6.1 software^62^ was utilized to clone the reverse translated vaccine peptide sequence into a pET-28a (+) expression plasmid vector, ensuring precise and effective production.

## Results

### Screening for B-cell epitopes

Upon employing the Kolaskar and Tongaonkar algorithm to examine the *S. parasitica* proteins SpHtp1, SpHtp3, and SPRG 19320, a total of 48 distinct linear epitopes for B-cells were identified.

### Prediction of MHC class I and II compatible epitopes

The employment of NetMHC 4.0 yielded a substantial number of 9-mer epitopes compatible with MHC class I molecules. Among them, a meticulous selection process identified 72 epitopes exhibiting strong binding characteristics, which were subsequently subjected to further analysis. Similarly, utilizing the NetMHCII 2.3 tool, a diverse range of 13-mer epitope sequences was generated and evaluated based on their binding affinity to MHC class II receptors. From this screening, the top 2% of epitopes displaying robust binding properties were chosen, resulting in the identification of 89 such epitopes.

### Designing the vaccine candidate

Following the selection process, the derived epitopes underwent antigenicity analyses using the VaxiJen 2.0 server against the target (Supplementary Tables S2–S4). From these analyses, highly antigenic epitopes were carefully selected. Utilizing suitable linker sequences these epitopes were then linked to one another, as well as to the immunogenic 50S ribosomal protein L7/L12 adjuvant. To complete the process, the RoseTTAFold algorithm was employed to design a coordinated structure for the vaccine.

### Stereochemical and physiochemical validation of the vaccine

**Figure 2:**
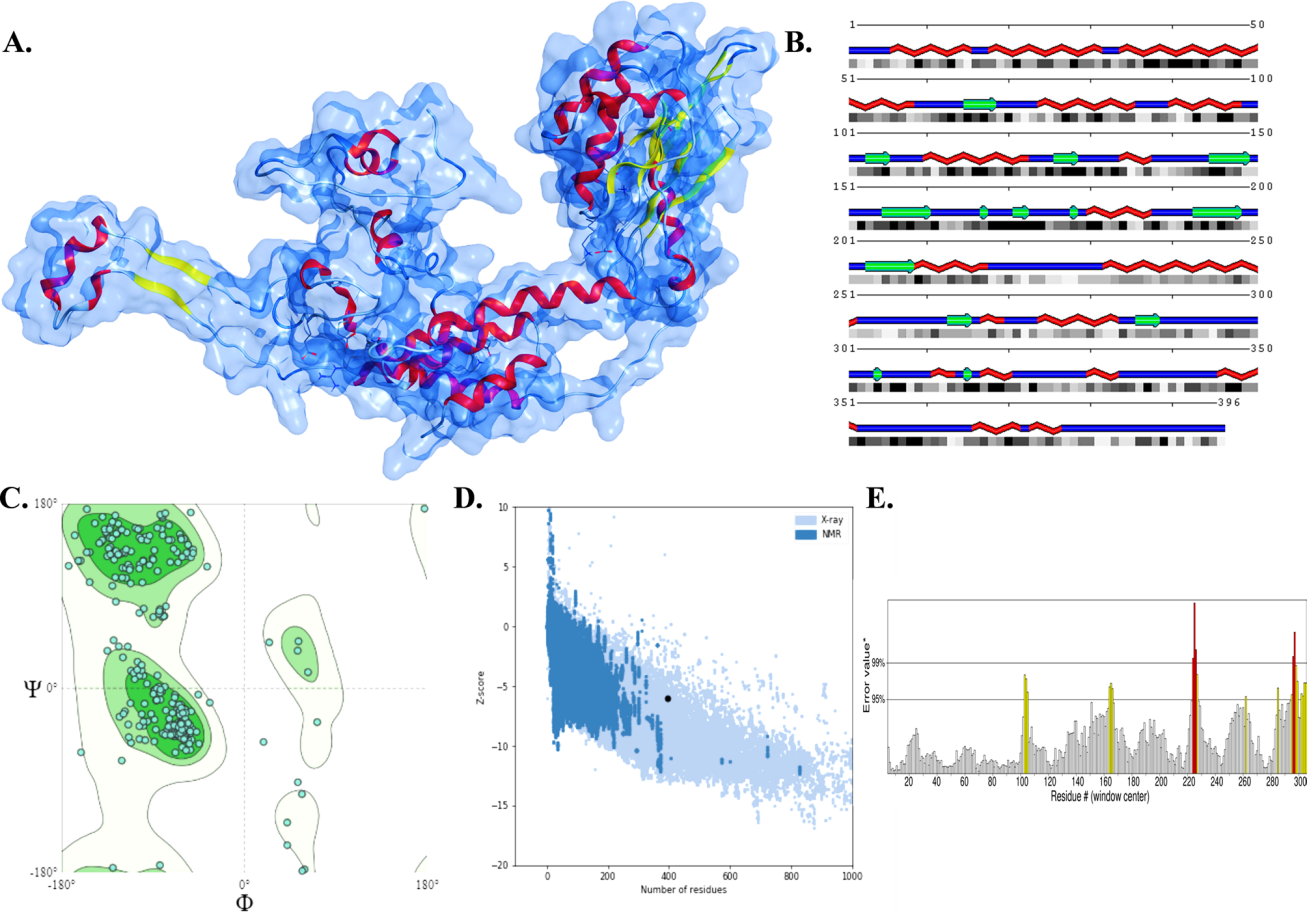
(**A**) The cartoon tertiary structure of the designed vaccine beneath the molecular surface. (**B**) The secondary structure of the vaccine. (**C**) The Ramachandran plot for the polypeptide, that is also validated by (**D**) Z-score plot as well as the (**E**) ERRAT plot.

It is crucial to assess the stereochemical quality of the model using various parameters. The SAVES 6.0 server yielded an ERRAT score of 90.814, while the vaccine demonstrated 92.39% Ramachandran-favored residues. Both results indicate a high-quality modelling performance. Additionally, the ProSA analysis displayed a Z-score of − 6.04, suggesting that the model closely aligns with X-ray-determined crystal structures (Fig. 2). Regarding the physical characteristics of the structure, it weighs 41.76 kDa and exhibits stability with an instability index of 29.12. This stability is further supported by an aliphatic index of 93.94. Moreover, the GRAVY (Grand average of hydropathy) value of − 0.30 indicates compatibility with the hydrophilic nature of the cytosol. It is evenly reflected by the solubility score of 0.564, as determined by the Protein-Sol server. Furthermore, the vaccine has a half-life of 30 h in mammalian reticulocytes, over 20 h in yeast, and more than 10 h in *E. coli*, indicating its durability in cellular environments (Supplementary Table S1). Additionally, when subjected to the ElliPro algorithm, the vaccine structure revealed the presence of eight conformational B-cell epitopes with scores ranging from 0.5 to 0.852 (Supplementary Fig. S1). This finding highlights the superior antigenic capacity of the vaccine, complementing its linear B-cell epitopes. In a nutshell, the model demonstrates the robust stereochemical quality and favourable characteristics, suggesting its efficacy and potential as an antigen.

### Molecular interactions of the vaccine with immune receptors

**Figure 3:**
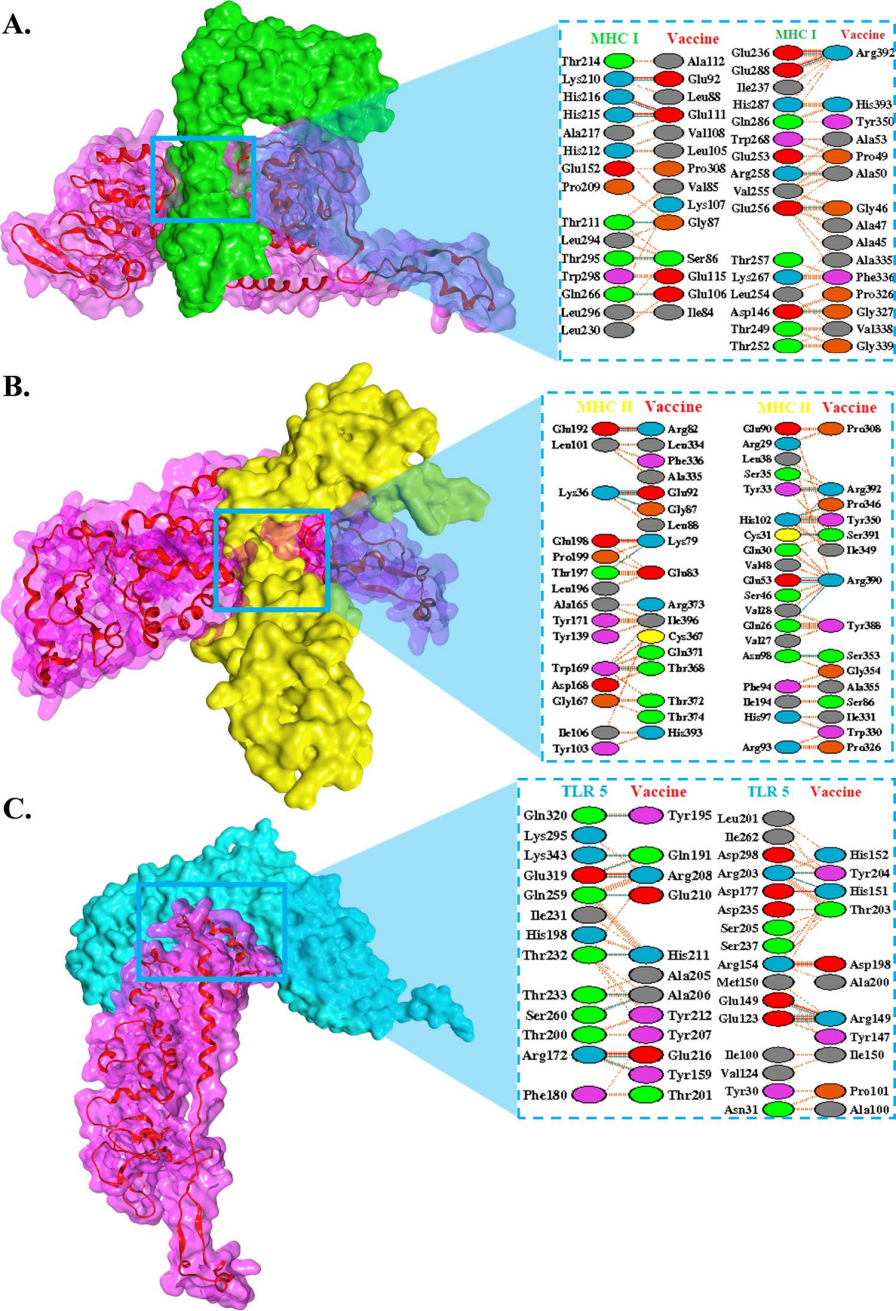
Shows the interacting residues in relation to the zoomed-out configuration of the vaccine when bound to (**A**) piscine HLA-A*02:01:01:01 MHC class I receptor, (**B**) piscine DAB1*07:01 MHC class II receptor, and also (**C**) piscine TLR 5 receptor.

To investigate the immunostimulatory potential of the vaccine, we employed molecular docking techniques to analyze its binding to immune receptors. ClusPro revealed that the TLR5 receptor binds to the vaccine with a score of − 947.2 kJ/mol. Wherein, the vaccine employs several of its residues including Arg149, Arg208, Aso198, Tyr212, Tyr207, and Glu216 to interact with Asp177, Arg172, Glu149, Glu319, Met150, Ile262, etc. on the fish receptor. Similarly, the vaccine peptide exhibited binding to the common fish MHC class I receptor, HLA-A*02:01:01:01, with a score of − 1035.4 kJ/mol. This interaction involved residues like Lys210, Val255, Glu288, Glu236, His212, Leu230, and others on the receptor, which primarily interacted with Arg392, Phe336, Ala50, Leu105, Gly46, Val85, Pro346, Pro326, and Leu88 on the vaccine. Moreover, ClusPro analysis demonstrated that the vaccine binds to the fish MHC class II receptor, DAB1*07:01, with a score of − 1227.6 kJ/mol. The binding interaction is orchestrated, primarily, by Trp169, Val28, Ile106, His102, Glu53, Phe94, Leu101, Gln26, Tyr103, Glu192, Ser35 on the MHC II receptor and Arg390, Arg82, Tyr350, Arg392, Thr368, Lys79, Ile331, Pro326, Pro346, Tyr388, Ser391, Phe336, etc. on the vaccine (Fig. 3, Table 1, Supplementary Fig. S2).

**Table 1:** Interaction analysis among vaccine and the immune receptors.

Interaction parameters		TLR5	MHC class I	MHC class II
Key interacting residues	Immune receptor	Asp177, Arg172, Glu149, Glu319, Met150, Ile262, Arg154, Thr233, Thr232, Val124, Leu201, Thr200	Lys210, Val255, Glu288, Glu236, His212, Leu230, Thr249, Leu254, Trp268	Trp169, Val28, Ile106, His102, Glu53, Phe94, Leu101, Gln26, Tyr103, Glu192, Ser35, Ile194, Tyr33, Cys31
	Vaccine	Arg149, Arg208, Asp198, Tyr212, Tyr207, Glu216, Ala206, His151, Tyr204, Pro101, Thr203, Ile150, His211, Ala205	Arg392, Phe336, Ala50, Leu105, Gly46, Val85, Pro346, Pro326, Leu88, Gly339	Arg390, Arg82, Tyr350, Arg392, Thr368, Lys79, Ile331, Pro326, Pro346, Tyr388, Ser391, Phe336, Pro308, Trp372, Gly87, Ser353
Energy components from MM/PBSA calculations (in kJ/mol)	Van der Waals contribution	− 284.54	− 121.23	− 289.56
	Electrostatic energy	− 1208.8	− 623.08	− 1128.78
	Polar solvation free energy	1201.1	648.87	1254.31
	Non-polar solvation free energy	− 40.01	− 16.66	− 39.88
	Total binding free energy	− 204.32	− 112.1	− 203.9

### Molecular dynamics analysis

Molecular dynamics simulations have been immensely useful in directing experimental validation. It analyses conformational changes, stability changes and overall evolution of the complex systems when emulated under cytosol-like conditions, by allowing evaluation of parameters like RMSD, RMSF, Rg, SASA and hydrogen bonding.

**Figure 4:**
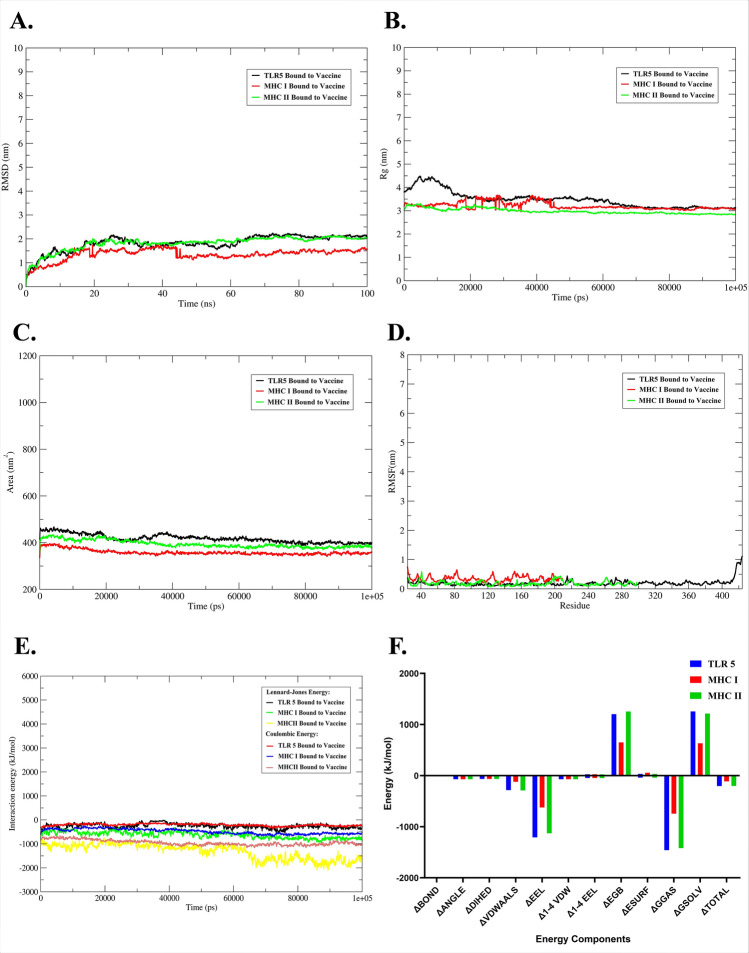
Different stability parameters derived from the 100 ns long molecular dynamics simulations studies of the complexes composed of vaccines bound to the immune receptors: (**A**) shows the plot for the RMSD in nm, (**B**) shows the radius of gyration expressed in nm, (**C**) gives the SASA, while (**D**) elaborates the residue wise RMSF. Similarly, (**E**) the Lennard–Jones and Coulombic energy values for the complexes. The free energy landscape of the complex interface was further revealed by the MM/PBSA calculations as described in (**F**).

RMSD serves as a crucial measurable parameter of receptor-ligand complex stability. Larger the RMSD observed; the lower the predicted stability of complex and vice-versa. In our MD-simulation study of complexes comprising of the vaccine polypeptide bound to TLR5, MHC-I and MHC-II, the average RMSD of the complexes were observed to be of values 0.18 nm, 0.6 nm and 0.98 nm, for the Vaccine-MHC II complex, Vaccine-TLR5 complex and Vaccine-MHC I complex, respectively (Fig. 4A). The lower values of RMSD are suggestive of the Vaccine-MHC II possessing more stability than the Vaccine-TLR5 complex and Vaccine-MHC I complex.

The compactness of the complex protein structure is defined by Rg, and complexes with lower Rg values indicate more compact structures with strong interactions. The average Rg throughout the trajectory in our study was found as 2.9 nm, 3.4 nm and 3.9 nm, for the Vaccine-MHC II complex, Vaccine-TLR5 complex and Vaccine-MHC I, respectively (Fig. 4B). The Rg values reiterated the indications of RMSD analyses and indicated the robust binding strength of the Vaccine-MHC II complex. Although the Vaccine-TLR5 complex exhibits minimal fluctuations in the initial 20 ns, it recovers to natural stability as does the Vaccine-MHC I complex.

SASA is a direct measurement of the surface area involved in interaction with the solvent during MD Simulation. An increment in SASA would denote expansion of the structural backbone of the complex protein. Our study revealed SASA values as—359.2 nm^2^, 394.7 nm^2^ and 418.1 nm^2^ for Vaccine-MHC I complex, Vaccine-MHC II, and Vaccine-TLR5, respectively (Fig. 4C).

The RMSF parameter is studied in MD Simulation to understand the main chain flexibility and its fluctuations at the residue level. In our study, the complexes of vaccine bound to MHC II, MHC I, and TLR5 show minimal fluctuations (Fig. 4D) throughout the structures and the RMSF values were observed between 0.19 and 0.47 nm. The Coulombian and Leonard–Jones interaction energies were observed to be highly negative in all the studied complexes of vaccine and selected immune receptors, reflecting their strong associations (Fig. 4E). The overall results of the MD simulations (Table 2) indicated towards stable and consistent complexes in the emulated micro-environmental conditions.

**Table 2:** Stabilization parameters derived from the molecular dynamics studies of complexes composing vaccine and immune receptors.

Stability component	TLR5	MHC class I	MHC class II
Average radius of gyration (in nm)	3.47243361	3.9611419	2.9804318
Average solvent accessible surface area (in nm^2^)	418.181035	359.207495	394.704818
Root mean square fluctuation (in nm)	0.19819277	0.35564403	0.47328561
Root mean square deviation (in nm)	0.60977221	0.98636141	0.18459453

### Binding strength

Calculation of binding free energy presents a crucial parameter in deciphering the molecular recognition process. In this study, we have used the MM/PBSA method which is considered as a very reliable and efficient approach in calculating binding free energy among protein–protein or protein–ligand systems. Figure 4F and Table 1 decomposes the energy components and visualizes the contribution of different interactions to the total binding free energy. The computed values follow a trend, − 204.32 kJ/mol <  − 203.9 kJ/mol <  − 112.1 kJ/mol for Vaccine-TLR5, Vaccine-MHC II, and Vaccine-MHC I complexes, respectively, indicating the vaccine’s capacity to offer strong interactions with the immune receptors.

### Immunostimulatory dynamics of the vaccine

**Figure 5:**
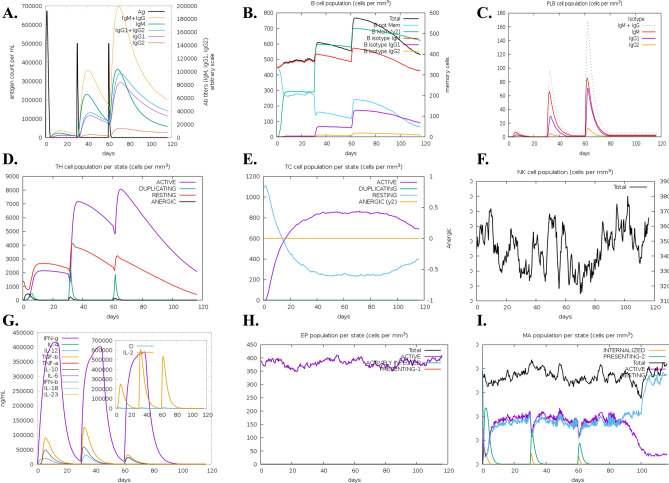
The 120-day long immune simulations composed of three consecutive vaccine doses separated by 30 days. It revealed potential efficacy of the vaccine in the host, as can be visualised by the titers of immunoglobulins and the immunocomplexes after vaccination as in (**A**) Whereas, (**B**) shows levels of B-cell population after vaccination. (**C**) Plots levels of plasma B-cell after vaccination. (**D**) Levels of helper T-cell cell population after vaccination. (**E**) Levels of cytotoxic T cells population after vaccination. (**F**) Levels of NK cell population after vaccination. (**G**) Concentration of cytokines and interleukins after vaccination. (**H**) Epithelial cell levels and (**I**) shows the levels of MA population after vaccination.

The utilization of immune simulation techniques is instrumental in comprehending the immune kinetics associated with the vaccine. Through the 120-day long simulation, significant findings have emerged as shown in Fig. 5. The initial dose triggered a pronounced immune response, characterized as a robust primary response. Subsequent doses administered on the 30th and 60th days evoked responses that surpassed the intensity of the primary response by multiple folds. This potent secondary response was observed through markedly elevated levels of IgM and IgG antibodies, as well as an increase in various immune cells present in the serum. Moreover, each subsequent dose contributed to faster antigen clearance. In the context of specific immune cell populations, the cytotoxic T cell population exhibited a peak of 1155 cells/mm^3^ on day 11, while the active cell population within this subset reached its maxima of 820 cells/mm^3^ on the 30th day. Following this peak, there was a gradual reduction in these cell populations from the 90th day onward. Additionally, the adaptive response demonstrated a parallel increase in tandem with the rise in the B-cell population, particularly the memory B-cells. The peaks observed were 700 cells/mm^3^ for memory B-cells and 580 cells/mm^3^ for IgM isotype B cells. Memory cells play a crucial role in regulating the prevention of *Saprolegnia* infection or re-infection in fish through their ability to self-memorize upon encountering pathogens. Furthermore, the administration of the vaccine was found to elevate other central regulators of the immune system, including cytokines, interleukins, and natural killer cells. These results underscore the designed vaccine as a potent candidate for eliciting a powerful and consistent immune response to combat fish pathogens.

### In silico cloning of the design

**Figure 6:**
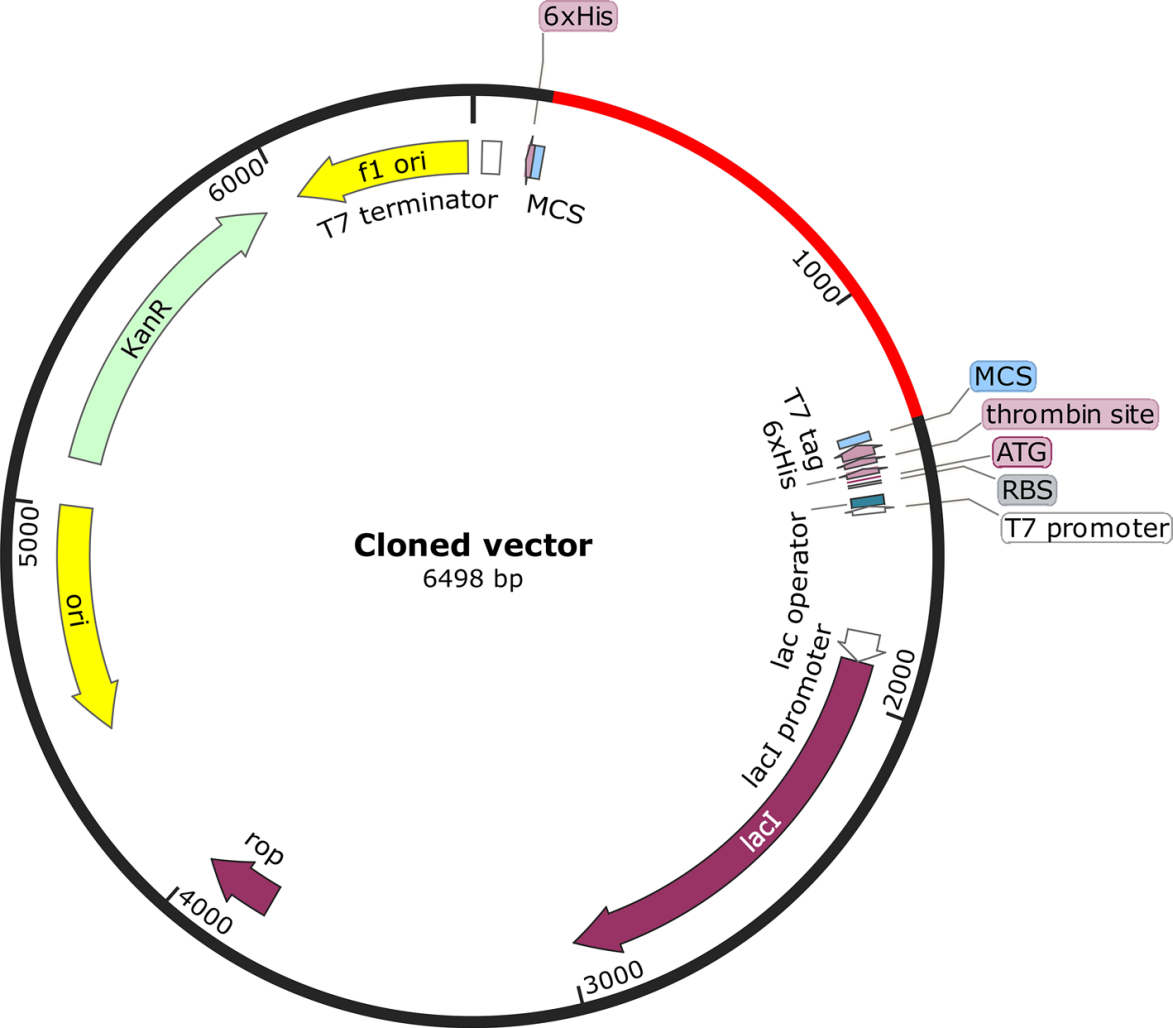
The cloned map of the vaccine cDNA sequence cloned into a pET-28a(+) plasmid for further expression in bacterial systems.

Efficient and rapid manufacturing of the vaccine candidate represents a crucial milestone in our endeavour to attain herd immunity and potentially eradicate *S. parasitica* as a prominent fish pathogen from various habitats. This is realized through the strategic cloning of the vaccine codons into meticulously optimized expression vectors. To ensure optimal processivity and expression within *E. coli* systems, the vaccine peptide sequence underwent a meticulous reverse translation process on the *E. coli* K12 codon table. CAI of the sequence was found to be 1.0. The index quantifies the extent to which the codon usage in a sequence aligns with the codon usage bias of the host organism. With its values spanning from 0 to 1, a CAI of 1.0 signifies ideal adaptation for our vaccine sequence. Moreover, the sequence exhibited a GC content of 52.52%, indicating favourable conditions for expression in *E. coli*. The optimal range for GC bases in this context typically falls between 30 and 70%. Subsequently, utilizing the SnapGene software, the vaccine sequence was finally inserted into a highly efficient pET-28a (+) expression plasmid vector (Fig. 6).

## Discussion

Saprolegniasis caused by *S. parasitica* has a profoundly detrimental impact on fish health, overall populations, and the global aquaculture-based economy. Hitherto, no safe and effective control regimen exists against this pathogen. The present research is designed to address this gap by developing a novel vaccine that targets key proteins involved in the infection process. The first vaccine developed by employing reverse vaccinology was the Meningococcal B Vaccine^63^ in early 2000, since then it has proved to be revolutionary to the way the vaccines are designed^64,65^. This study harnesses similar approaches and focuses on three key proteins of SpHtp1, SpHtp3, and SPRG19320 based on the crucial roles they play in the critical early stages of infection and their significant abundance in the secretome of *S. parasitica*. To elicit robust adaptive immunity in conjunction with innate responses, the proteins were subjected to B-cell epitope scanning. Subsequently, successful prediction of MHC-I and MHC-II epitopes was achieved using the NetMHC 4.0 and NetMHCII 2.3 servers, respectively. Nevertheless, highly antigenic B and T-cell epitopes were then carefully selected and connected with appropriate peptide linkers to construct the vaccine candidate. Additionally, the 50S ribosomal protein L7/L12 adjuvant was conjugated at the N-terminal end with a suitable linker to enhance immunogenicity at the cellular level. The coordinate structure of the vaccine was developed using the RoseTTAFold algorithm and subsequently validated using the ExPASy Structure Assessment Tool, ProSA, and ERRAT. The analysis demonstrated that the model consisted of 92.39% Ramachandran-favored residues, indicating a high-quality stereochemical configuration. Also, it achieved an ERRAT score of 90.814 and a Z-score of − 6.04, further confirming its reliable structural integrity. Interestingly, the ElliPro program determined 8 different conformational epitopes on analysis of the vaccine structure. Molecular docking studies, conducted on the ClusPro supercomputer server, revealed that binds with not only TLR5 with a score of − 947.2, but also with MHC class I and MHC II, scoring − 1035.4 and − 1227.6, respectively. Moreover, molecular dynamics studies were employed to gain insights regarding the cytosolic stability of the complexes hence formed. The dynamic trajectories of the simulations were, further, used for calculating the binding strength among the vaccine and the immune receptors by MM-PBSA methods. Finally, through a 120-day immune simulation, significant findings have emerged. The initial dose triggered a strong primary immune response. Subsequent doses administered on days 30 and 60 elicited even more powerful secondary responses, with significantly elevated levels of antibodies and various immune cells in the serum. Notably, each successive dose accelerated the pathogen clearance capacity of the host and induce strong immunogenic memory. In a nutshell, the vaccine was able to bind to the piscine TLR molecules as well as the MHC proteins with strong binding affinity. The interactions with the immune receptors were analysed by molecular dynamics simulations and found to be stable and versatile suggesting strong immunogenicity of the vaccine, further, this was correlated with the findings of the immune simulation studies. Finally, we were able to conduct successful in silico cloning of the vaccine suggesting efficient expression in *E. coli,* systems for large scale manufacture. Nevertheless, the study demands further in vitro and in vivo experimentation to examine its behaviour in physiological conditions. That said, the developed vaccine candidate projects a potent prophylactic option against saprolegniasis.

## Conclusion

*Saprolegnia parasitica* is essentially noxious to the global aquaculture industry, yet, we lack any safe and effective treatment option against it. To address this pathogen and develop a prophylactic measure, we analyzed its three key proteins SpHtp1, SpHtp3, and SPRG19320 to determine safe and immunogenic epitopes that can induce humoral as well as cell-mediated immunity. Subsequently, a potent multi-epitope vaccine candidate was designed. It was predicted to be non-allergenic and had suitable physiochemical characteristics. Molecular docking studies complemented with molecular dynamics simulations indicated the vaccine’s ability to form strong and stable interactions with the immune receptors. Nonetheless, it was fascinating to find the potential efficacy of the vaccine design in eliciting strong immune responses against the pathogen, as the immune simulations suggest. The construct being a promising solution to combat saprolegniasis, this study warrants in-depth in vitro as well as in vivo investigation for evaluating the efficacy of the vaccine in native conditions.

## Supplementary Information

Supplementary Information.

## Data Availability

Data is with the authors and will be provided on request through the corresponding author.
