# Author Correction: Immunoinformatics approaches in developing a novel multi-epitope chimeric vaccine protective against *Saprolegnia parasitica*

**DOI:** 10.1038/s41598-025-89657-y

**Published:** 2025-02-13

**Authors:** Abhigyan Choudhury, Pawan Kumar, Hiba‑Allah Nafdi, Khalid S. Almaary, Gezahign Fentahun Wondmie, Ajit Kumar, Mohammed Bourhia

**Affiliations:** 1 Independent Researcher, Asansol, West Bengal 713 305 India; 2 Toxicology and Computational Biology Group, Centre for Bioinformatics, Maharshi Dayanand University, Rohtak, 124 001 India; 3 Department of Food Science, Faculty of Agricultural and Food Sciences, Laval University, Quebec City, QC 2325G1V 0A6 Canada; 4 Department of Botany and Microbiology, College of Science, King Saud University, P. O. Box 2455, 114 51 Riyadh, Saudi Arabia; 5 Department of Biology, Bahir Dar University, Po.Box 79, Bahir Dar, Ethiopia; 6 Department of Chemistry and Biochemistry, Faculty of Medicine and Pharmacy, Ibn Zohr University, 700 00 Laayoune, Morocco; 7 Laboratory of Chemistry-Biochemistry, Environment, Nutrition, and Health, Faculty of Medicine and Pharmacy, University Hassan II, B. P. 5696 Casablanca, Morocco

Correction to: *Scientific Reports* 10.1038/s41598-024-52223-z, published online 27 January 2024

In the original version of this Article, Abhigyan Choudhury was incorrectly affiliated with the ‘Department of Animal Science, Kazi Nazrul University, Asansol, West Bengal, 713 340, India.’. The correct Affiliation is listed below.

- “Independent Researcher, Asansol, West Bengal, India 713 305.”

The original Article has been corrected.

