# Supplementary material for: Immunoinformatics approaches in developing a novel multi-epitope chimeric vaccine protective against Saprolegnia parasitica
Source: Sci Rep. 2024 Jan 27;14:2260. doi: 10.1038/s41598-024-52223-z (PMC10817918; doi:10.1038/s41598-024-52223-z)
Supplement: Supplementary file 1 — Supplementary Information. [file 41598_2024_52223_MOESM1_ESM.docx]

**SUPPLEMENTARY MATERIAL**

**Immunoinformatics approaches in developing a novel multi-epitope chimeric vaccine protective against *Saprolegnia parasitica***

Abhigyan Choudhury*^1^, Pawan Kumar^2^, Hiba-Allah Nafidi^3^, Khalid S. Almaary^4^, Gezahign Fentahun Wondmie*^5^, Ajit Kumar*^2^ and Mohammed Bourhia^6^

^1^ Department of Animal Science, Kazi Nazrul University, Asansol - 713 340, West Bengal, India

^2^ Toxicology and Computational Biology Group, Centre for Bioinformatics, Maharshi Dayanand University, Rohtak, 124 001, India

^3^ Department of Food Science, Faculty of Agricultural and Food Sciences, Laval University, 2325 Quebec City, QC G1V 0A6, Canada

^4^ Department of Botany and Microbiology, College of Science, King Saud University, P. O. Box 2455, Riyadh 114 51, Saudi Arabia

^5^ Department of Biology, Bahir Dar University, Po.Box 79, Bahir Dar, Ethiopia

^6^ Department of Chemistry and Biochemistry, Faculty of Medicine and Pharmacy, Ibn Zohr University, Laayoune 700 00, Morocco

***Correspondence:** akumar.cbt.mdu@gmail.com (AK); [abhigyan6531@gmail.com](mailto:abhigyan6531@gmail.com) (AC); [resercherfent@gmail.com](mailto:resercherfent@gmail.com) (GFW)


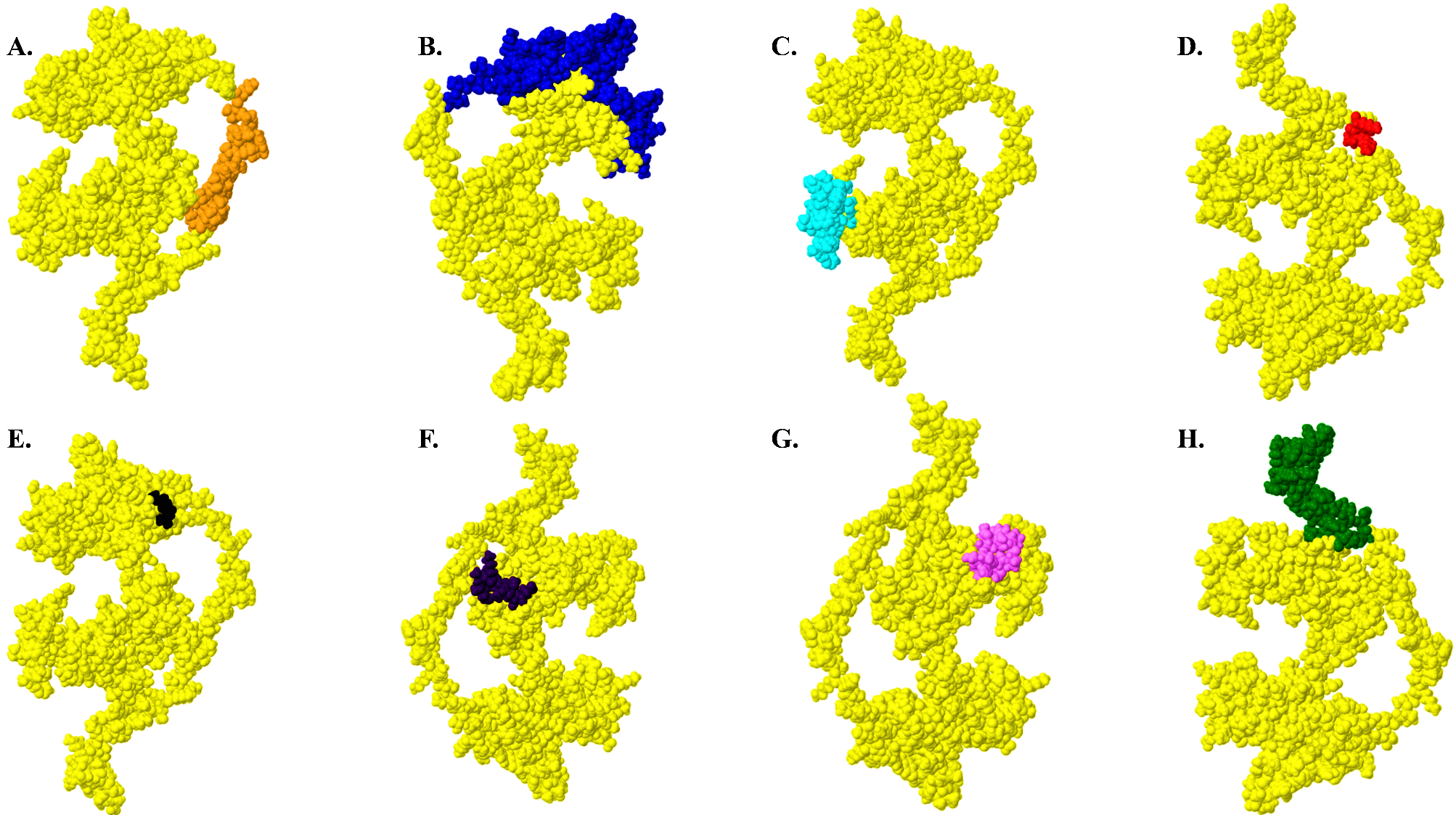


**Supplementary Figure S1.** The different conformational B-cell epitopes on the vaccine, as determined by the ElliPro algorithm.

**
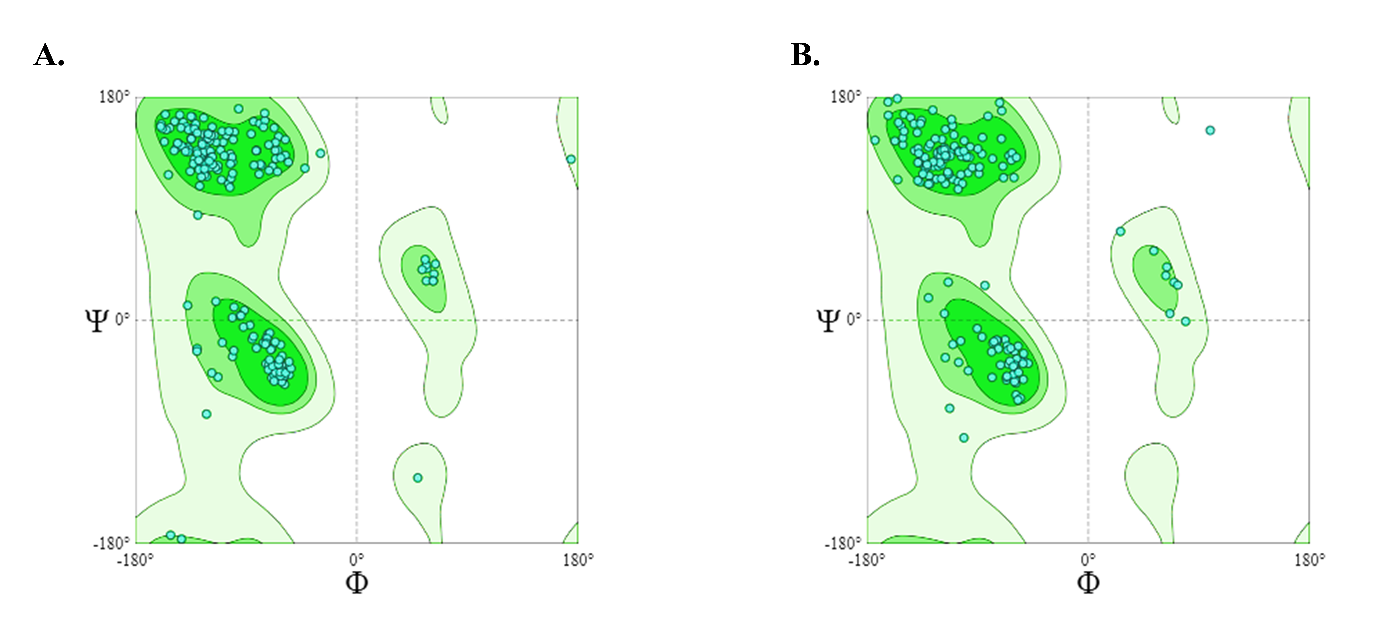
**

**Supplementary Figure S2. A.** Ramachandran plot for the modelled MHC class I HLA-A*02:01:01:01 protein having 98.57% residues in favoured region. While, **B.** is the Ramachandran plot of MHC class II DAB1*07:01 protein with 95.65% favoured residues.

**Supplementary Table S1.** Physiochemical properties of the vaccine candidate

|  | **Values** |
| --- | --- |
| **Molecular Weight** | 41.76 kDa |
| **Half-life** | 30 hrs in mammalian reticulocytes |
| **Instability Index** | 29.12 |
| **Aliphatic index** | 93.94 |
| **GRAVY** | -0.30 |
| **Solubility (Protein-Sol)** | 0.564 |
| **Antigenicity (VaxiJen)** | 0.8283 |
| **Allergenicity (AllerTOP 2.0)** | Non-Allergen |
| **Conformation Epitopes** | 8 |
| **The estimated half-life in mammalian RBCs** | 30 hours |
| **The estimated half-life in yeast** | >20 hours |
| **The estimated half-life in *E. coli.*** | >10 hours |

**Supplementary Table S2.** Antigenic B-cell epitopes derived from the three pathogenic proteins named SpHtp1, SpHtp3 and SPRG 19320

| **Epitope** | **Length** | **VaxiJen Score** |
| --- | --- | --- |
| **Host targeting protein 1 (SpHtp1)** | | |
| HHPLTLAALCVVLHESLGAA | 20 | 0.5799 |
| RLEHYRIAEI | 10 | 0.9692 |
| APTVAPT | 7 | 0.6633 |
| **Host targeting protein 3 (SpHtp3)** | | |
| VPVWIPILAFAVGLGLGLLIPHLQKP | 26 | 1.1805 |
| GKVVSVT | 7 | 0.7864 |
| QLRVAGVDCP | 10 | 1.0799 |
| QDQVVSFKL | 9 | 1.1723 |
| SGAVYGG | 7 | 0.8322 |
| **SPRG 19320** | | |
| SAECGVGEQVRFL | 13 | 3.0263 |
| KCDVTVDFR | 9 | 1.9366 |
| PCAVDCQVSA | 10 | 1.4071 |
| SAQSCMQV | 8 | 1.2510 |
| GKVCNLY | 7 | 1.2540 |
| QQPCSTS | 7 | 1.2197 |
| CDECTGV | 7 | 1.0527 |
| CGSCADIVSAF | 11 | 0.8820 |

**Supplementary Table S3.** Antigenic MHC class I epitopes derived from the three *Saprolegnia* proteins named SpHtp1, SpHtp3 and SPRG 19320

| **Epitope** | **VaxiJen Score** |
| --- | --- |
| **Host targeting protein 1 (SpHtp1)** | |
| MRIHHPLTL | 1.3171 |
| EQATTGNSV | 2.4451 |
| TLAALCVVL | 0.6467 |
| **Host targeting protein 3 (SpHtp3)** | |
| KLTETTLQL | 0.8357 |
| VTDGDTIRV | 4.1294 |
| VVSFKLLMK | 1.1069 |
| RVNIWSLDK | 1.0584 |
| EVPVWIPIL | 2.0521 |
| TLRGKVVSV | 0.9747 |
| TIRVRHVPW | 1.1164 |
| IRVRHVPWL | 1.2702 |
| MKDQYSRAV | 0.9053 |
| LEVPVWIPI | 2.0726 |
| **SPRG 19320** | |
| TTDSQTCAV | 1.2341 |
| RTGSATYLY | 1.2078 |
| ACDPATGTY | 1.2821 |
| YMPPICSKL | 1.0773 |
| SPACNGGKV | 1.0410 |
| APAINGGTC | 1.1664 |
| YVRSRHVPI | 2.4813 |
| MRLSLAIAT | 1.1504 |
| SRTEVTPAL | 0.6105 |
| YEVAQELGL | 1.6766 |
| AECGVGEQV | 3.0602 |

**Supplementary Table S4.** Antigenic MHC class II epitopes derived from the three pathogenic proteins named SpHtp1, SpHtp3 and SPRG 19320

| **Epitope** | **VaxiJen Score** |
| --- | --- |
| **Host targeting protein 1 (SpHtp1)** | |
| AALCVVLHESLGA | 0.7011 |
| ALCVVLHESLGAA | 0.7155 |
| LAALCVVLHESLG | 0.6844 |
| LCVVLHESLGAAQ | 0.7142 |
| HSNNVARLEHYRI | 0.9588 |
| SNNVARLEHYRIA | 0.8405 |
| NNVARLEHYRIAE | 0.5837 |
| NVARLEHYRIAEI | 0.5876 |
| **Host targeting protein 3 (SpHtp3)** | |
| ILAFAVGLGLGLL | 0.7943 |
| LAFAVGLGLGLLI | 1.0418 |
| PILAFAVGLGLGL | 1.2975 |
| GDTIRVRHVPWLA | 1.2093 |
| DGDTIRVRHVPWL | 1.6765 |
| ILAFAVGLGLGLL | 0.7943 |
| PILAFAVGLGLGL | 1.2975 |
| VPWLANGDGDFKG | 0.7660 |
| PWLANGDGDFKGK | 1.3333 |
| AFAVGLGLGLLIP | 1.1021 |
| IPILAFAVGLGLG | 1.3687 |
| VYYGSWAAPMNVS | 1.3417 |
| NIYRQSGAVYGGL | 1.0548 |
| VSFKLLMKDQYSR | 0.8495 |
| **SPRG 19320** | |
| RRTINVDYPAQNG | 0.6962 |
| SRRTINVDYPAQN | 0.6479 |
| DANYVRLTVALRQ | 0.7554 |
| ATGTYVRSRHVPI | 1.5026 |
| TGTYVRSRHVPID | 0.9470 |
| SCMQVSTTTMLYP | 0.6237 |
| MRLSLAIATVILT | 0.7412 |
| SNKSYALAAAATG | 0.9265 |
| CTGVQTRTRKVLI | 0.9322 |
| YVRLTVALRQNQC | 0.5247 |
| GTYVRSRHVPIDA | 0.8940 |
| MRLSLAIATVILT | 0.7412 |
| AREVLQQQVNCGK | 0.5877 |
| ECTGVQTRTRKVL | 0.9359 |
